# Movement patterns and habitat use of tiger sharks (*Galeocerdo cuvier*) across ontogeny in the Gulf of Mexico

**DOI:** 10.1371/journal.pone.0234868

**Published:** 2020-07-15

**Authors:** Matthew J. Ajemian, J. Marcus Drymon, Neil Hammerschlag, R. J. David Wells, Garrett Street, Brett Falterman, Jennifer A. McKinney, William B. Driggers, Eric R. Hoffmayer, Christopher Fischer, Gregory W. Stunz

**Affiliations:** 1 Harbor Branch Oceanographic Institute, Florida Atlantic University, Fort Pierce, Florida, United States of America; 2 Coastal Research and Extension Center, Mississippi State University, Biloxi, Mississippi, United States of America; 3 Mississippi-Alabama Sea Grant, Ocean Springs, Mississippi, United States of America; 4 Rosenstiel School of Marine & Atmospheric Science, University of Miami, Causeway, Miami, Florida, United States of America; 5 Abess Center for Ecosystem Science & Policy, University of Miami, Miami, Florida, United States of America; 6 Department of Marine Biology, Texas A&M University at Galveston, Galveston, Texas, United States of America; 7 Department of Wildlife & Fisheries Sciences, Texas A&M University, College Station, Texas, United States of America; 8 Quantitative Ecology & Spatial Technologies Laboratory, Mississippi State University, Starkville, Mississippi State, United States of America; 9 Department of Wildlife, Fisheries, and Aquaculture, Mississippi State University, Starkville, Mississippi State, United States of America; 10 Louisiana Department of Wildlife and Fisheries, New Orleans, Louisiana, United States of America; 11 NOAA Fisheries, Southeast Fisheries Science Center, Mississippi Laboratories, Pascagoula, Mississippi, United States of America; 12 OCEARCH, Park City, Utah, United States of America; 13 Harte Research Institute for Gulf of Mexico Studies, Texas A&M University-Corpus Christi, Corpus Christi, Texas, United States of America; Department of Agriculture, Water and the Environment, AUSTRALIA

## Abstract

The tiger shark (*Galeocerdo cuvier*) is globally distributed with established coastal and open-ocean movement patterns in many portions of its range. While all life stages of tiger sharks are known to occur in the Gulf of Mexico (GoM), variability in habitat use and movement patterns over ontogeny have never been quantified in this large marine ecosystem. To address this data gap we fitted 56 tiger sharks with Smart Position and Temperature transmitting tags between 2010 and 2018 and examined seasonal and spatial distribution patterns across the GoM. Additionally, we analyzed overlap of core habitats (i.e., 50% kernel density estimates) among individuals relative to large benthic features (oil and gas platforms, natural banks, bathymetric breaks). Our analyses revealed significant ontogenetic and seasonal differences in distribution patterns as well as across-shelf (i.e., regional) and sex-linked variability in movement rates. Presumably sub-adult and adult sharks achieved significantly higher movement rates and used off-shelf deeper habitats at greater proportions than juvenile sharks, particularly during the fall and winter seasons. Further, female maximum rate of movement was higher than males when accounting for size. Additionally, we found evidence of core regions encompassing the National Oceanographic and Atmospheric Administration designated Habitat Areas of Particular Concern (i.e., shelf-edge banks) during cooler months, particularly by females, as well as 2,504 oil and gas platforms. These data provide a baseline for future assessments of environmental impacts, such as climate variability or oil spills, on tiger shark movements and distribution in the region. Future research may benefit from combining alternative tracking tools, such as acoustic telemetry and genetic approaches, which can facilitate long-term assessment of the species’ movement dynamics and better elucidate the ecological significance of the core habitats identified here.

## Introduction

Understanding movement patterns and dynamic habitat use for widely ranging species is a significant challenge in the marine environment. This is especially true for highly migratory sharks, which often traverse regional, national, and international boundaries, thus encountering a broad range of environmental and anthropogenic stressors [1,2,3,4]. Alarmingly, more than one-fourth of highly migratory sharks are characterized by the International Union for the Conservation of Nature as Critically Endangered, Endangered or Vulnerable [5]. Clearly, a thorough understanding of highly migratory shark movement patterns and habitat preferences is urgently needed for developing comprehensive management and conservation strategies [6–8].

Despite the highly mobile nature of many sharks, these animals have been shown to exhibit extended residence within certain oceanographic features characterized by high productivity [8]. These habitats can be dynamic and include meso-scale eddies [9,10] and ocean-estuarine interfaces [11], while others can be fixed and more structurally complex, such as reefs, ridges, seamounts and banks [12–14]. However, the remoteness and ephemeral nature of some of these features often requires sophisticated tools to reveal individual use patterns by free-ranging sharks. Fortunately, satellite telemetry has emerged as a powerful tool that has increased our ability to assess habitat preferences and movement patterns for highly mobile species, including sharks [15]. Continued increases in battery life, coupled with decreases in the size and cost of transmitters, have resulted in a more complete understanding of dynamic habitat use for otherwise elusive species [16]. In fact, recent collaborative efforts have provided estimates of space use for several species of sharks spanning much of the globe [8]. Despite these advances, gaps remain in our understanding of the spatial dynamics of many highly mobile sharks, including variability across ontogeny and over their ranges.

The tiger shark (*Galeocerdo cuvier*) is a globally distributed, highly mobile species with established coastal and open-ocean movement patterns that have been revealed via satellite telemetry [17]. Previous studies have noted variable patterns of space use in tiger sharks, ranging from resident to highly migratory behavior [17]. The majority of this work occurred around the Hawaiian Islands in the eastern central Pacific Ocean where tiger sharks typically display site fidelity to core islands but also move between islands for foraging purposes [18–21]. Similar patterns have been observed off the Galápagos Islands, where tiger sharks can have highly resident behavior within the marine reserve but often traverse deep waters outside the reserve and visit areas off continental South America [22]. In the western North Atlantic Ocean, adult tiger sharks tagged near Bermuda show considerable basin-wide connectivity, integrating multiple ecosystems (temperate to tropical); however, these findings were almost exclusively based on male sharks [4]. Similarly, tiger sharks (predominately female) tagged off south Florida and the northern Bahamas appear to exhibit associations with the Gulf Stream, presumably due to the high productivity, and thus food availability, in this current system [23,24]. By combining tracks from mostly adult female tiger sharks tagged in Florida and the Bahamas with remotely sensed environmental data, Calich et al. [25] predicted large areas of suitable habitat off the southeast United States, including the Gulf of Mexico (GoM). However, actual use of the predicted suitable habitat by tiger sharks remains unknown as does the importance of these habitats for males and juveniles.

The GoM is a highly productive marginal sea, home to a diverse community of coastal sharks [11,26,27], including tiger sharks [28]. To date, satellite telemetry has been used to describe the movement patterns and habitat preferences of multiple GoM shark species including scalloped hammerhead (*Sphyrna lewini*) [29,30], dusky sharks (*Carcharhinus obscurus*) [31] shortfin makos (*Isurus oxyrinchus*) [32], and whale sharks (*Rhincodon typus*) [33,34]. While all life stages of tiger shark are known to occur in the GoM [11,25,28], detailed habitat use has never been quantified. This is striking as the GoM faces numerous anthropogenic stressors [35–37], complex tri-national management [38,39], and indications of size reductions in recreational landings for large sharks [40,41]. Additionally, the potential for ontogenetic and sex-specific habitat partitioning by tiger sharks remains unknown in these waters. Although the species does not use discrete nurseries for parturition in the GoM, it has been suggested that the nearshore waters of the region are important for neonates [28,42] and by extension, could also serve a critical role for gravid females. A recent study demonstrated the capacity of tiger sharks to traverse tri-national boundaries within the GoM, particularly during the winter [39]. However, the former study did not include: 1) an assessment of sex-based differences in distribution patterns, 2) quantification of movement rates, and 3) potential interactions with large-scale habitat features, all of which have been identified as information-deficient areas in need of additional research [17]. Therefore, the goals of this study were to address these aforementioned knowledge gaps for tiger sharks in the GoM.

## Methods

### Ethics statement

This study was carried out in strict accordance with the Animal Welfare Act and other Federal statutes and regulations relating to animals. The protocol was approved by the Institutional Animal Care and Use Committee at Texas A&M University-Corpus Christi (Animal Use Protocol: #08-18) and permitted under a Letter of Acknowledgement (SHK-LOA-14-08) from the National Marine Fisheries Service, Highly Migratory Species Division. All efforts were made to minimize animal suffering during collection and tagging procedures.

### Animal collection and tagging

Tiger sharks (n = 56; 32 ♀, 24 ♂) were captured and tagged throughout the northern GoM from 2010 to 2018, spanning shelf waters from south Texas to south Florida (Table 1). Sharks were collected using bottom longline (BL, n = 32), drum-line (DL, n = 17), and hook-and-line (HL, n = 7) gears. The BL and DL captured individuals were retrieved from the water and secured to a platform along either the stern or gunwale of the vessel. The HL-caught sharks remained submerged following capture and secured alongside the vessel with the leader and a tail rope. Body length measurements included pre-caudal length (PCL, cm), fork length (FL, cm) and stretched total length (STL, cm). In those cases when only STL was recorded, *FL* was estimated using the equation derived from our capture data:

FL(cm)=0.8338*STL(cm)−7 (1)

The sex of each individual was determined and maturity state was assigned for males via established methods such as physical examination of clasper rotation and calcification [43]. Following Branstetter et al. [44], in cases where maturity could not be assessed, sharks were considered mature at 265 and 258 cm FL for females and males, respectively. A conventional mark-recapture tag was attached on the trunk of each individual at the base of the first dorsal fin and a Smart Position and Temperature (SPOT) transmitting tag (Wildlife Computers, Inc.) secured behind the anterior margin of the first dorsal fin.

**Table 1:** Collection information and tag performance of individuals tagged in study.

Study ID	PTT ID	FL (cm)	STL (cm)	Sex	LAT	LON	Gear	Model	MTPD	Tagging Date	DAL	Locations	Transmit Days
Shark-1	34020	210	263	M	26.37	-81.98	DL	258	250	5/25/2010	42	41	17
Shark-2	34107	210	256	F	26.37	-81.98	DL	258	250	5/25/2010	206	5	2
Shark-3	33992	161	203	F	26.37	-81.98	DL	258	250	5/26/2010	33	16	8
Shark-4	34021	197	241	F	26.37	-81.98	DL	258	250	5/26/2010	25	48	13
Shark-5	34029	205	255	F	26.37	-81.98	DL	258	250	5/26/2010	191	80	31
Shark-6	55495	235	295	F	26.37	-81.98	DL	258	250	6/09/2010	128	178	91
Shark-7	55494	198	250	F	26.37	-81.98	DL	258	250	6/10/2010	95	64	36
Shark-8	68477	178	200	M	26.37	-81.98	DL	258	250	10/29/2010	127	87	44
Shark-9	68471	197	245	F	24.70	-80.85	DL	258	250	1/29/2011	27	7	4
Shark-10	68554	*335*	403	F	26.86	-79.04	DL	258	250	2/9/2011	194	338	143
Shark-11	120899	235	290	F	25.35	-82.07	BL	196	250	8/14/2012	29	78	22
Shark-12	120901	213	260	M	29.41	-84.01	BL	196	250	8/22/2012	22	12	6
Shark-13	120881	230	282	F	29.40	-84.01	BL	258	250	8/23/2012	25	11	4
Shark-14	120900	210	250	F	28.56	-91.34	BL	196	250	9/18/2012	125	13	6
Shark-15	120894	134	174	M	27.92	-84.17	BL	196	250	9/27/2012	102	195	48
Shark-16	130985	248	289	F	24.70	-80.85	DL	258	250	6/01/2013	290	307	116
Shark-17	120880	*156*	192	M	29.34	-84.07	BL	258	250	8/22/2013	27	241	26
Shark-18	120906	185	271	F	28.91	-92.97	BL	257	250	9/10/2013	53	342	49
Shark-19	120908	223	231	F	28.91	-92.97	BL	257	250	9/10/2013	58	275	35
Shark-20	133723	204	294	F	24.89	-80.98	DL	258	250	11/06/2013	253	131	46
Shark-21	129957	180	230	M	26.34	-81.95	DL	258	250	11/13/2013	60	138	28
Shark-22	111551	139	180	F	25.01	-81.00	DL	258	250	11/21/2013	29	80	13
Shark-23	120885	*192*	239	F	30.18	-88.95	BL	258	250	7/7/2014	50	413	48
Shark-24	141585	248	311	M	27.89	-96.42	HL	258	70	8/12/2014	189	92	45
Shark-25	141586	233	282	F	27.90	-96.43	HL	258	70	8/12/2014	695	155	110
Shark-26	120877	265	320	F	28.63	-94.77	BL	258	250	9/12/2014	228	659	116
Shark-27	132414	178	224	M	28.57	-90.36	BL	257	250	9/16/2014	13	42	11
Shark-28	132430	151	200	F	28.30	-90.76	BL	258	250	9/23/2014	43	267	43
Shark-29	120907	221	271	M	28.31	-92.84	BL	257	250	9/27/2014	129	235	44
Shark-30	146598	197	245	M	25.75	-80.17	DL	258	250	3/15/2015	233	265	107
Shark-31	132416	173	217	M	29.79	-86.31	BL	257	250	3/18/2015	48	121	34
Shark-32	132413	212	263	M	29.78	-88.07	BL	257	250	4/5/2015	51	110	34
Shark-33	151867	244	301	F	29.94	-87.57	BL	258	300	8/10/2015	26	40	11
Shark-34	151868	245	300	M	29.94	-87.57	BL	258	300	8/10/2015	37	16	4
Shark-35	151866	107	136	M	29.79	-87.61	BL	258	300	8/11/2015	15	22	8
Shark-36	151875	109	142	F	29.87	-87.54	BL	258	300	8/11/2015	N/A	N/A	N/A
Shark-37	151876	107	138	F	29.87	-87.54	BL	258	300	8/11/2015	6	9	4
Shark-38	151877	210	258	M	29.86	-87.58	BL	258	300	8/11/2015	79	32	10
Shark-39	151878	136	167	F	29.87	-87.54	BL	258	300	8/11/2015	14	42	8
Shark-40	151879	140	183	M	29.87	-87.54	BL	258	300	8/11/2015	24	20	6
Shark-41	151880	119	155	F	29.86	-87.58	BL	258	300	8/11/2015	10	4	1
Shark-42	151413	*263*	320	M	27.91	-96.44	HL	257	70	11/5/2015	63	119	41
Shark-43	151420	250	303	F	27.74	-96.24	DL	257	70	11/10/2015	415	406	151
Shark-44	160310	*228*	280	M	29.89	-87.75	BL	258	250	5/12/2016	240	508	72
Shark-45	160312	141	178	M	29.68	-88.17	BL	258	250	5/13/2016	N/A	N/A	N/A
Shark-46	160313	266	360	M	30.14	-87.55	BL	258	250	7/20/2016	38	127	18
Shark-47	160314	230	277	M	30.04	-87.60	BL	258	250	8/4/2016	19	179	17
Shark-48	160309	160	200	M	29.60	-88.13	BL	258	250	8/19/2016	N/A	N/A	N/A
Shark-49	159825	*102*	136	F	27.88	-93.82	HL	258	70	3/20/2017	174	89	45
Shark-50	159826	210	260	F	27.88	-93.82	HL	258	70	3/20/2017	44	35	18
Shark-51	160311	189	240	F	29.47	-88.22	BL	258	250	4/25/2017	50	42	10
Shark-52	151432	185	229	F	26.14	-97.15	HL	258	70	6/9/2017	17	27	9
Shark-53	169319	250	305	M	29.99	-87.77	BL	258	250	6/19/2017	21	23	2
Shark-54	153522	203	254	F	27.12	-97.01	HL	257	70	8/5/2017	N/A	N/A	N/A
Shark-55	169320	215	269	M	29.71	-87.59	BL	258	250	9/18/2017	161	138	21
Shark-56	19687	298	339	F	25.78	-80.10	DL	258	250	10/25/2018	297	432	165

Summary information is presented for individual tiger shark size (fork length – FL; stretched total length – STL), tagging location, collection method (gear) and transmitter performance. Individuals with estimated fork length are italicized. Latitude (LAT) and longitude (LON) are in decimal degrees For collection gear, BL = Bottom long-line, DL = drum-line, and HL = hook-and-line. DAL = Days at Liberty. MTPD = maximum transmissions per day.

### Data processing and analyses

Animal position estimates were downloaded from Argos satellites (CLS America, Inc.). For analyses, we included all position estimates of class B or higher (A, 0, 1, 2, and 3) and excluded Z class transmissions [45]. The following metrics were calculated for each individual: 1) days at liberty (days from release to last transmission), 2) number of locations, and 3) transmit days (number of days with at least a single position). Linear regressions were performed on these metrics to assess the potential effect of shark size on the length of the transmission period. A correlated random walk state-space model was used to regularize daily positions using the FoieGras package (https://github.com/ianjonsen/foieGras) in R (R Core Team, Vienna, Austria) [46]. This model also provided estimates of east-west and north-south velocity, from which a resultant overall velocity metric, or rate of movement (ROM), was computed using the Pythagorean Theorem. For instances where intervals between consecutive position estimates exceeded 4 days, daily positions were not interpolated. Only data from individuals with at least 10 transmit days were included in statistical analyses (n = 38).

Underlying bottom depths were extracted in ArcMap from regularized position estimates using the ETOPO1 bathymetry raster data sets [47]. To facilitate general spatial and ontogenetic analyses of habitat use, FoieGras-based positions were assigned one of three underlying depth categories: 1) shelf (0 – 200 m), 2) slope (200 – 1000 m), and abyssal (>1000 m). Additionally, individual shark sizes were placed into three size bins to conceptualize distribution patterns by life stage: 1) small (<200 cm FL; n = 18), 2) medium (200 – 250 cm FL; n = 15), and 3) large (>250 cm FL; n = 5), reflecting immature, transitional, and mature sizes, respectively.

We used General Linear Models (GLMs) to examine the potential effects of the factors sex, season (winter, spring, summer, and fall), and region (shelf, slope, abyssal) on two response variables: 1) maximum ROM and 2) maximum underlying depth used by sharks. The GLMs for maximum ROM included separate two-way analyses of sex and season and sex and region. A three-way analysis (sex, season, and region) was not possible due to insufficient numbers of individuals (replicates) of a given sex during certain seasons or regions. The GLM for maximum underlying depth included a single two-way analysis of the factors sex and season; region was not used as a factor in this model since underlying depth was used to define region (see above). All GLMs used FL as a covariate in the model to control for effects of shark size. In cases where significant effects of factors were found, post-hoc comparisons were run using Tukey’s pairwise comparisons. Where necessary, both ROM and depth data were square-root transformed prior to analyses in order to meet assumptions of parametric statistics. All GLMs were run using Minitab 19.1.1 (Minitab LLC) with an α value of 0.05.

The regularized daily position estimates were used to build 50% and 95% kernel density estimates (KDEs) for each individual in R using the adehabitatHR package with the “href” bandwidth estimator. The resulting KDEs were plotted in ArcMap 10.3 (ESRI, Inc.) to identify overall distribution patterns as well as core areas of use. We used a general linear model to assess potential sex- and size-based differences in 50% and 95% KDEs, using transmission days and FL as covariates. Data were checked prior to analysis for normality (Shapiro-Wilk) and homogeneity of variances (Levene’s Test). Following Graham et al. [2], we considered the 50% KDE as core habitat use areas. Therefore, interactions between shark core habitat use areas and underlying habitat features (e.g. bathymetry, oil and gas structures, natural banks) was explored by examining the overlap of 50% KDEs with features of interest in ArcMap. Overlapping polygons were joined into a single feature class, with centroids (points) used to define the number of individual overlaps via the join tool. This polygon data set was then converted to a raster to facilitate extraction of values from underlying habitat features.

## Results

### Shark size distribution

**Fig 1:**
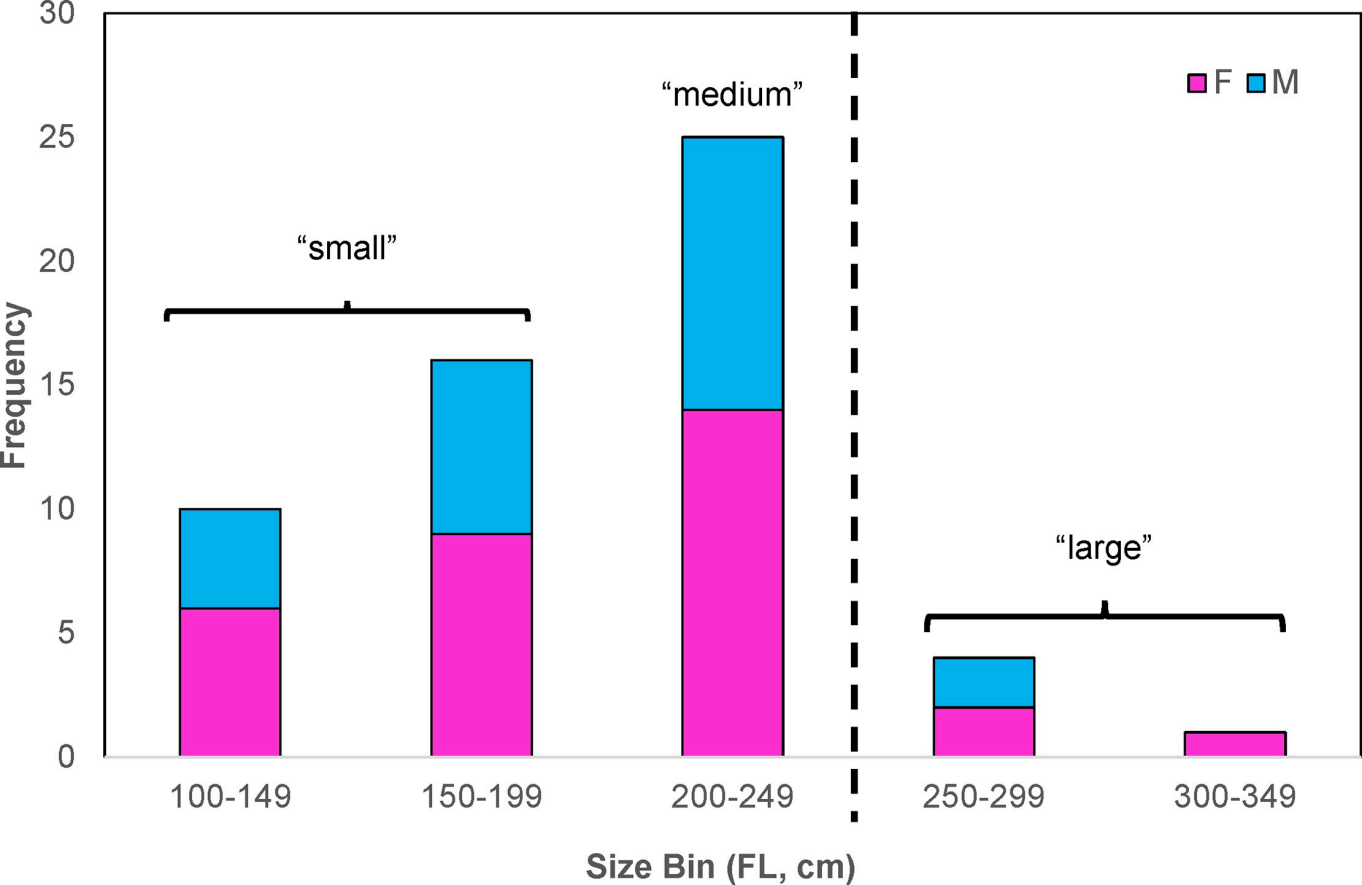
Frequency histogram of tiger shark sizes (fork length, FL) tagged in this study. Data are presented in 50 cm size bins. Sexes are represented by color (blue = male, pink = female). Vertical dotted line represents size-at-maturity break.

The relationship between FL and STL was strongly linear (*R^2^* = 0.95) and was used to estimate FL for individuals with missing data (n = 5). Shark size ranges were similar between males and females (**Fig 1**), with females ranging from 102–335 cm FL (mean ± s.d. = 200 cm ± 54 cm FL) and males ranging from 107–266 cm FL (mean ± s.d. = 198 ± 44 cm FL). Mean FL was not statistically different between sexes (two-sample t-test, *t* = -0.155, d.f. = 54, *P* = 0.877). Only five individuals were presumably mature at the time of tagging, which included three females (Shark-10, Shark-26, and Shark-56) and two males (Shark-42, Shark-46); as such, most of the individuals tagged were likely immature or sub-adult.

### Days at liberty and transmission days

**Fig 2:**
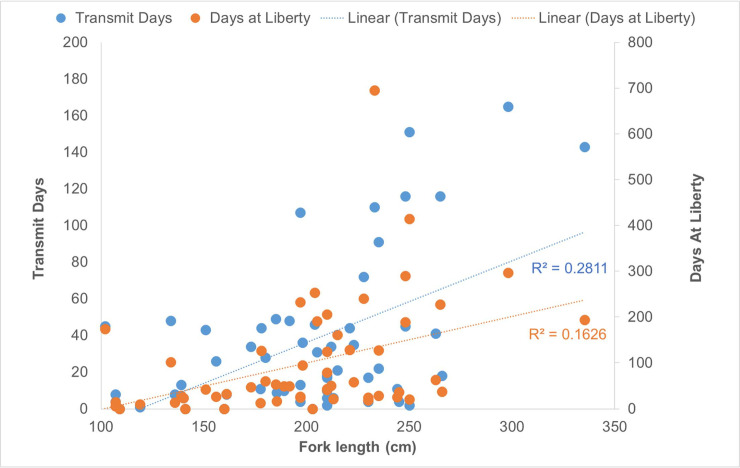
Scatterplot of track duration by shark size. Data are presented by transmit days (left axis, blue dots) and days at liberty (right axis, orange dots) by fork length (FL, cm). Linear regressions and r-squared values are indicated by dashed lines.

Days at liberty varied among individuals, ranging from 6 to 695 d (mean = 107.1 ± 125.1 d). Two individuals (both females) were tracked greater than 12 months: Shark-25 (233 cm FL at tagging; 695 d) and Shark-43 (250 cm FL at tagging; 415 d), both released off the Texas coastal bend region. The three next longest tracking durations all came from females (204−298 cm FL at tagging). Given these results, we ran linear regressions on days at liberty and transmit days using FL as a continuous predictor and sex as a categorical predictor. Regression analyses indicated a significantly positive impact of fork length on transmission days (*F*_1,51_ = 17.82; *P* < 0.0001; *R*^2^ = 0.28), and days at liberty (*F*_1,51_ = 8.22; *P* = 0.006; *R*^2^ = 0.10); however, this effect was independent of sex (*P* > 0.05). The scatterplot of these relationships suggested that they were driven by substantially higher transmit days and liberty for the medium to large size classes (i.e., > 200 cm FL; Fig 2).

### Movement patterns and distribution by size, sex, and season

**Fig 3:**
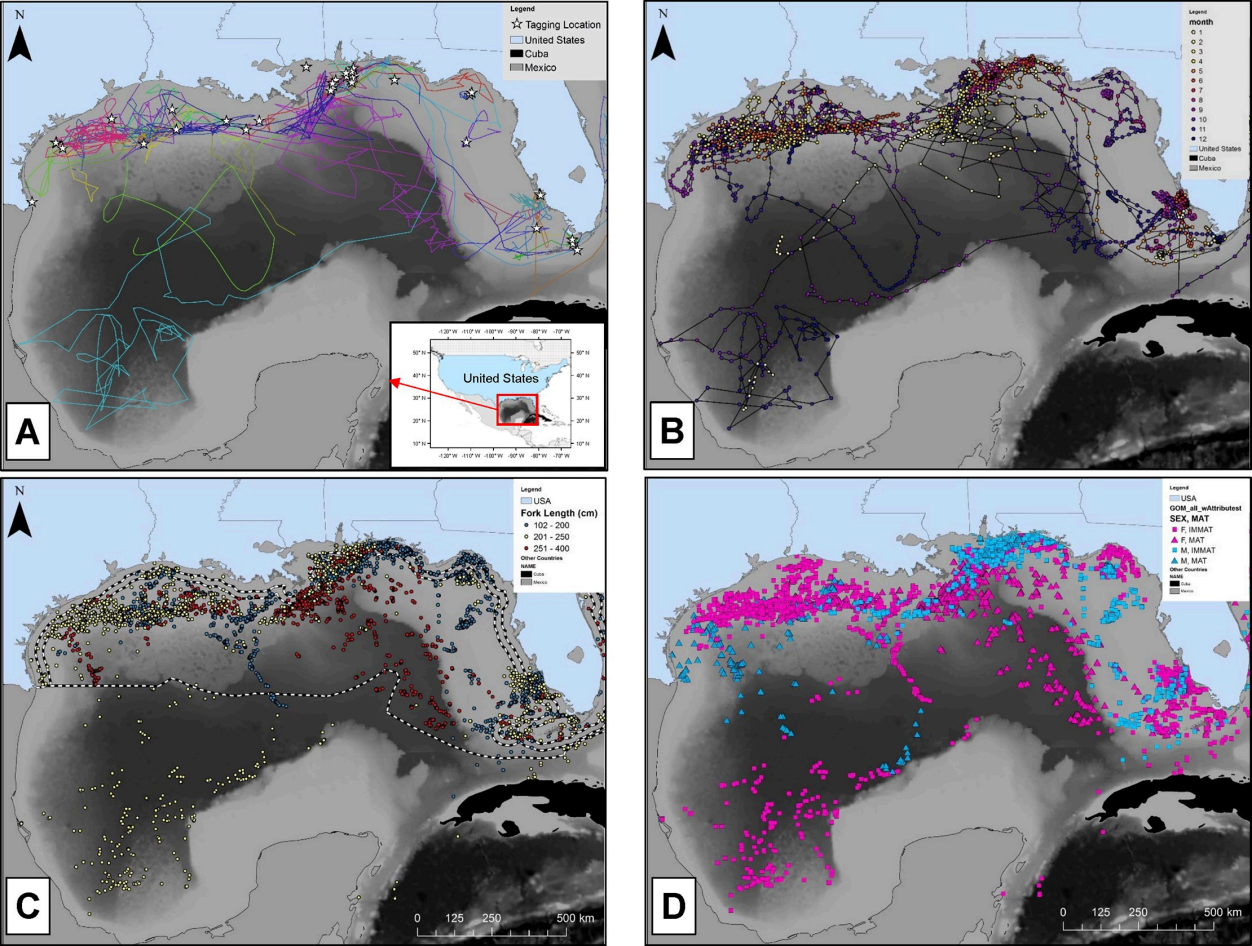
Maps of tiger shark tracks and distribution. A: Tag release locations (white stars) and individual tracks (colored lines) of tiger sharks fitted with SPOT transmitters from 2010 to 2018. Tracks are based on daily position estimates from the down-sampled data set. Inset map with red box delineates the Gulf of Mexico Large Marine Ecosystem. B: Tracks and positions displayed by month (color) to document seasonality of positions. C: Position estimates plotted by shark size (at time of tagging) category, small (100-200 cm FL, blue), medium (201-250 cm FL, yellow), and large (251-400 cm FL; red), and D: Position estimates based on maturity (squares = immature; triangles = mature) and sex (blue = males, pink = females).

Regularized daily position estimates (n = 5,513) were obtained for 52 of the 56 tagged sharks and were somewhat evenly distributed across small (n = 2,022), medium (n = 1,680), and large (n = 1,811) size classes. Tracks generated from these positions were variable; many were tightly coupled to the continental shelf edge, while others extended across the GoM basin (Fig 3A). In general, tracks across the basin appeared more directed, and became more circuitous as they approached the continental slope and shelf. Cross-basin movements were most apparent in late-fall through early winter (Fig 3B). All three size categories of sharks (small, medium, and large) occurred in waters overlying shelf, slope, and abyssal habitats. There was evidence of intermediate size classes of both sexes over the interior of the GoM, beyond the U.S. Exclusive Economic Zone in Mexican and Cuban waters; however, there appeared to be a general ontogenetic transition from inshore to offshore waters with size (Fig 3C). Male and female distributions overlapped throughout the GoM, with a dominance of mature individuals along the shelf-edge and slope habitats, and immature sharks along the nearshore region (Fig 3D).

**Fig 4:**
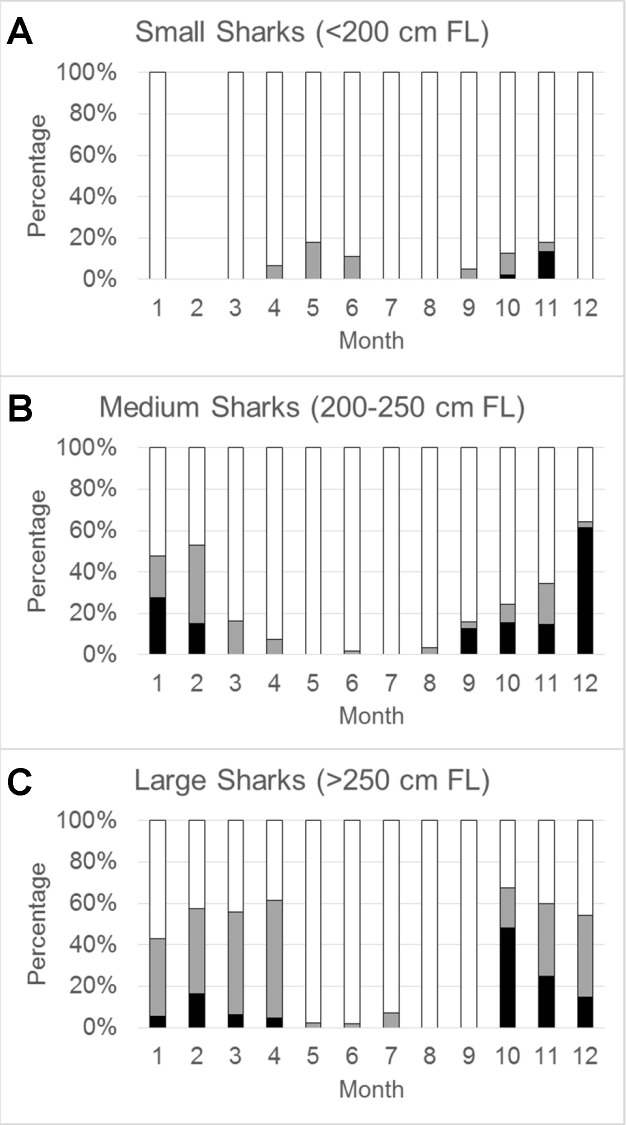
Relative distribution of positions by month for three size classes of tiger sharks across three regions. Data are pooled across individuals presented as 100% stacked bars per month, with colors representing different regions: continental shelf (white bars), continental slope (gray bars), and abyssal plain (black bars).

The relative proportional use of waters overlying the three habitat categories was variable by both time of year and size class (Fig 4). Small sharks (<200 cm FL; n=18) were detected from 0-335 km offshore (mean = 73.4 ± 65.2 km) and primarily found in shelf habitats throughout the year (91% of positions; Fig 4A). These smaller individuals were positioned nearshore along the Florida coast, particularly during the summer months (Fig 3C). That said, there was some evidence of slope water use from April to June, and again from September to November (7% of positions; Fig 4A). Relatively few positions were estimated from small sharks over abyssal waters in October and November (2% of total positions), and all came from a single individual. No small sharks transmitted in February. Medium-sized sharks (200-250 cm FL; n=15) had a similar distribution of positions (mean = 87.0 ± 72.1 km) as the smaller sharks, with most coming from shelf waters (77% of positions); however, there was a higher proportion of positions over slope waters (9% of positions) and abyssal waters (14% of positions). Medium-sized sharks were primarily positioned along shelf waters from May to August, and increased occupancy over deeper slope and abyssal waters through December. A transition from these waters overlying deep habitats to slope waters was evident in early winter to spring. Large individuals (>250 cm FL; n=6) ranged from 0-413 km offshore (mean = 113.2 ± 72.9 km) and had the least number of positions over shelf waters (59%) among size classes, and highest number of positions over slope waters (31%). Shelf habitats were primarily used by large sharks between May and September, after which a stark transition to slope and abyssal waters (10%) was evident for the majority of individuals, primarily from October through April.

### Rates of movement and depth use statistics

**Fig 5:**
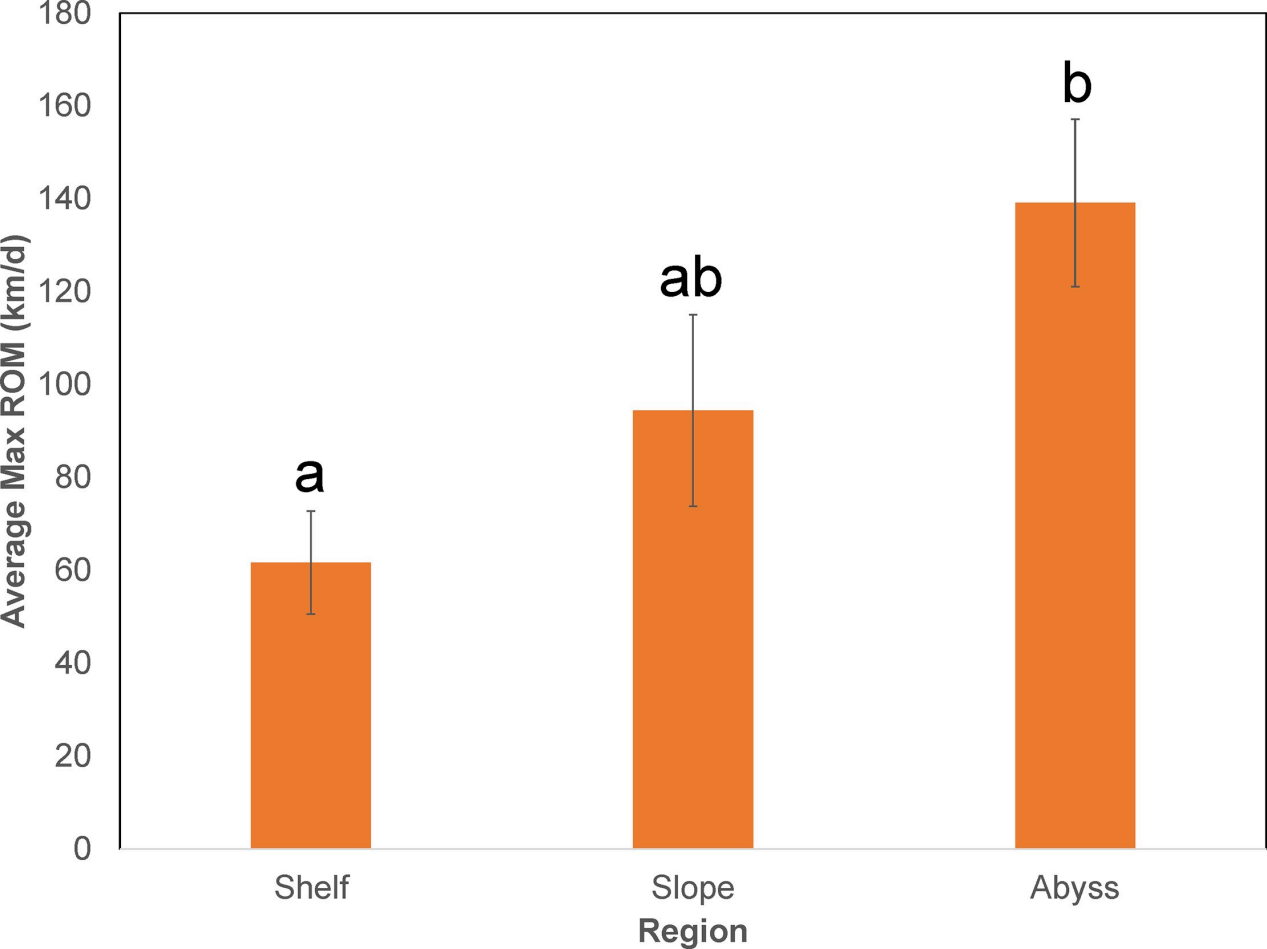
Vertical bar chart of average maximum rate rate of movement by region. Data are presented across continental zones in the GoM. Error bars represent standard errors of the mean and letters designate statistically significant groups as revealed by Tukey’s pairwise comparisons. Regions that share at least one letter are not significantly different from one another (i.e,. *P* > 0.05), whereas those that do not share a letter are statistically distinct (i.e., *P* < 0.05).

Regression analysis of maximum ROM by fork length showed a weak but significant positive linear relationship (*F*_1,38_ = 4.7; *P* = 0.036; *R*^2^ = 0.11). We therefore incorporated size into subsequent analyses that involved ROM. General linear models run on maximum ROM revealed a significant effect of the covariate size (*F*_1,69_ = 5.13; *P* = 0.027), but not season (*F*_3,69_ = 0.20; *P* = 0.897), sex (*F*_1,69_ = 3.24; *P =* 0.077), or the interaction of the two factors (*F*_3,69_ = 1.01; *P* = 0.395; Table 2). Analysis of square-root transformed maximum ROM found significant effects of region (*F*_2,59_ = 3.74; *P* = 0.030) and sex (*F*_1,59_ = 5.35; *P* = 0.025), but not for the interaction between the two factors (*F*_1,59_ = 1.80; *P* = 0.175) or the size covariate (*F*_1,59_ = 2.30; *P* = 0.135; Table 2). Pairwise comparisons showed that maximum ROM was significantly higher in waters above abyssal depths (average = 139.1 ± 37.8 km·d^-1^; compared to shelf waters (average = 61.7 ± 8.4 km·d^-1^;), but not slope waters (average = 94.5 ± 13.2 km·d^-1^; Fig 5). Furthermore, female max ROM (average = 98.7 ± 14.8 km·d^-1^) was significantly higher than males (average = 63.6 ± 8.8 km·d^-1^).

**Table 2:** General linear model results on square-root transformed maximum rate of movement.

Source	DF	Adj SS	Adj MS	F-Value	P-Value
FL	1	23.873	23.873	2.3	0.135
Region	2	77.608	38.804	3.74	****0.030****
Sex	1	55.436	55.436	5.35	****0.025****
Region*Sex	2	37.407	18.704	1.8	0.175
Error	53	549.672	10.371		
Total	59	757.655			

Results are shown for the factors size class, region, and the interaction between these two factors. Significant p-values are depicted in bold (P < 0.05).

**Fig 6:**
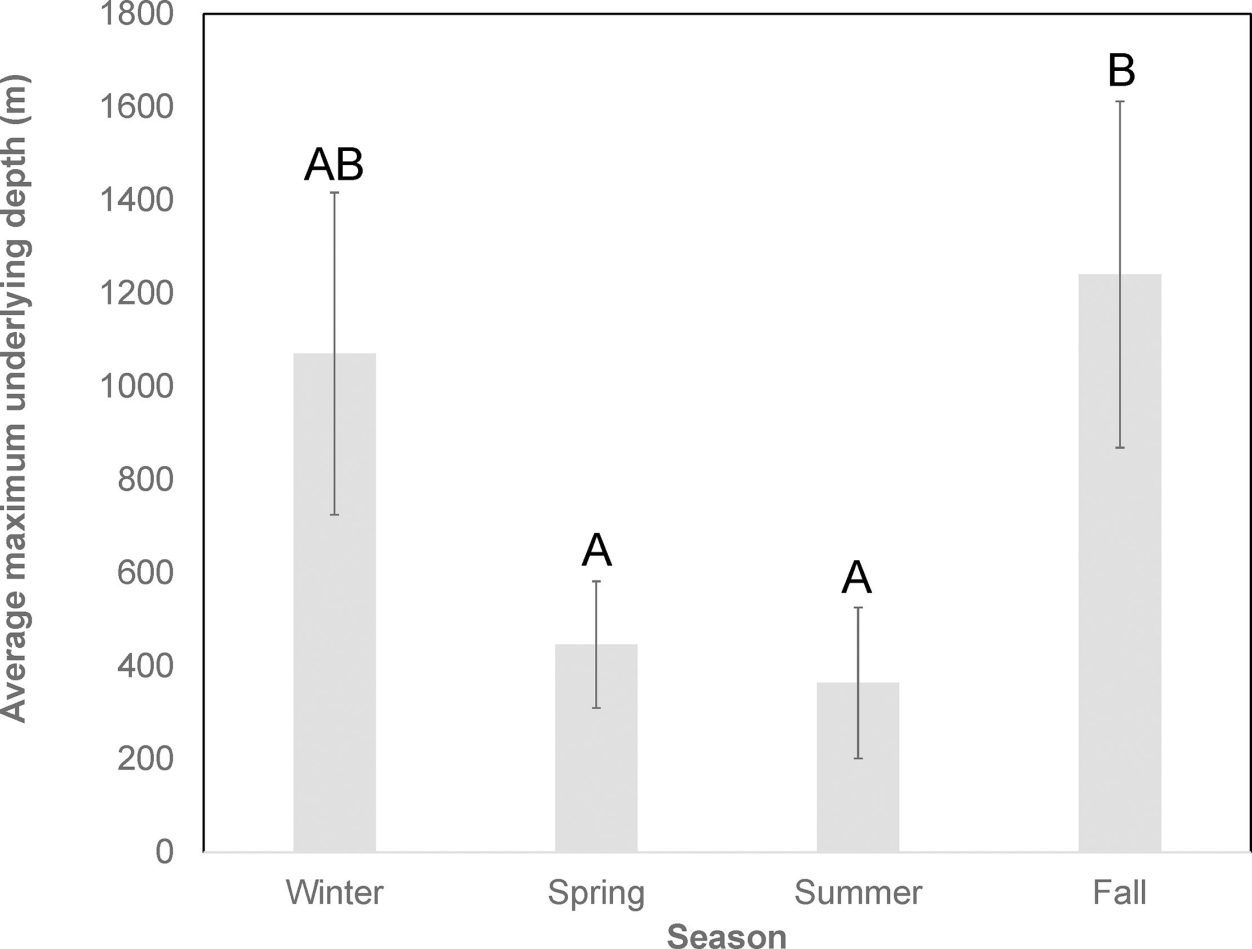
Vertical bar chart of average maximum underlying bottom depths by season. Mean values are individual averages. Error bars represent standard errors of the mean and letters designate statistically significant groups as revealed by Tukey’s pairwise comparisons. Seasons that share at least one letter are not significantly different from one another (i.e,. *P* > 0.05), whereas those that do not share a letter are statistically distinct (i.e., *P* < 0.05).

Average maximum underlying depth was significantly influenced by size (*F*_1,69_ = 6.98; *P* = 0.010) and season (*F*_3,69_ = 3.09; *P* = 0.034), but not sex (*F*_1.69_= 0.04; *P* = 0.022), or the interaction between sex and season (*F*_3,69_ = 0.26; *P* = 0.853; Table 3, Fig 6). Pairwise comparisons revealed that mean underlying depths during fall (mean = 1240 ± 372 m) were significantly greater than both spring (mean = 446 ± 136 m) and summer (mean = 363 ± 162 m), but not winter (mean = 1071 ± 346 m).

**Table 3:** General linear model results on maximum underlying depth used by sharks.

Source	DF	Adj SS	Adj MS	F-Value	P-Value
FL	1	7658344	7658344	6.98	****0.010****
Season	3	10161274	3387091	3.09	****0.034****
Sex	1	45146	45146	0.04	0.840
Season*Sex	3	860594	286865	0.26	0.853
Error	61	66899167	1096708		
Total	69	85641327			

Results are shown for the factors season, sex, and the interaction between these two factors. Fork length (FL) was used as a covariate in the model. Significant p-values are depicted in bold (P < 0.05).

### Kernel density estimates and core use areas

**Fig 7:**
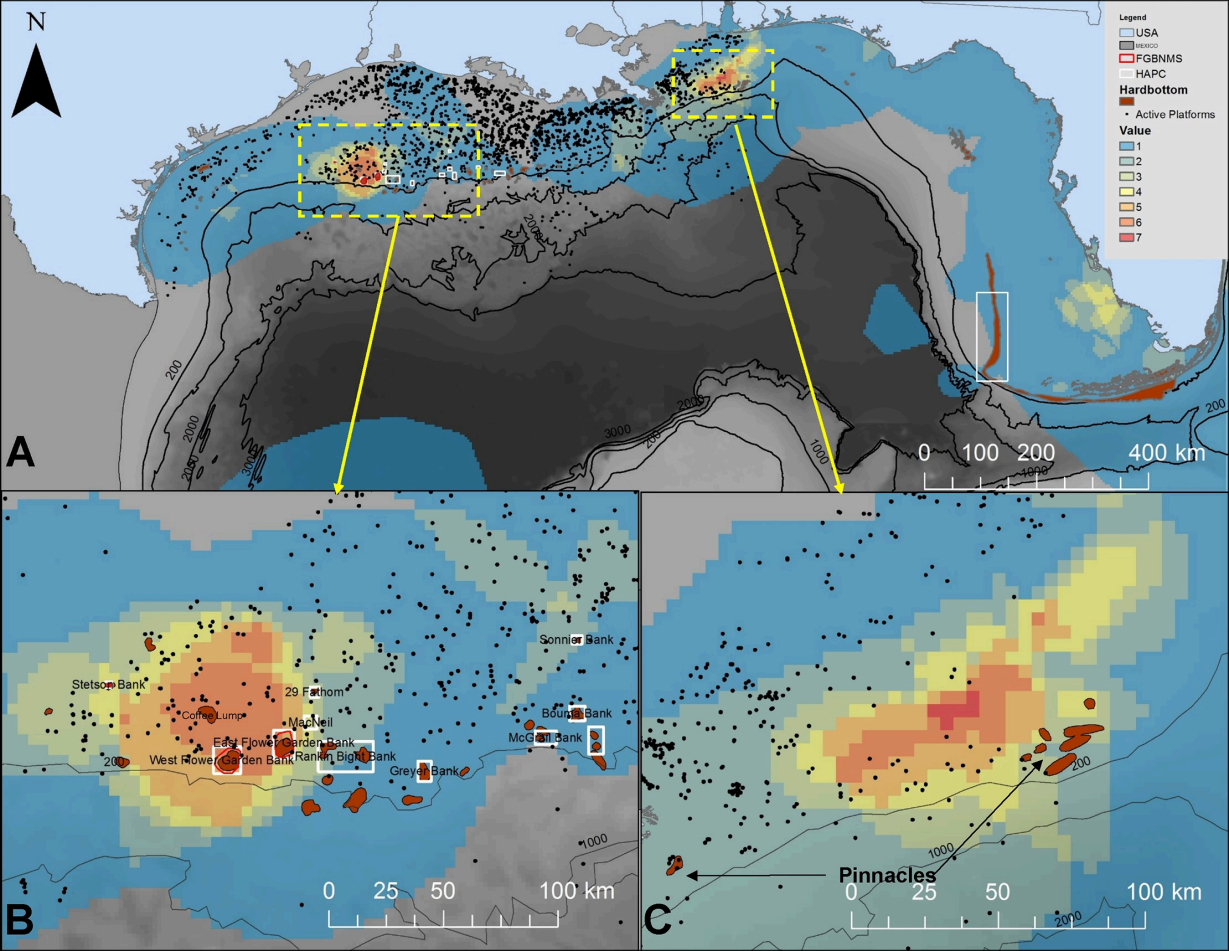
Map of core habitat use areas of tiger sharks in the GoM. Imagery represents results from 50% KDE overlap analysis relative to other features in the GoM. Overview map (A) shows KDE overlap relative to bathymetry (black lines) and large habitat features such as oil and gas platforms (black dots), National Oceanographic and Atmospheric Administration designated Habitat Areas of Particular Concern (white boxes) and benthic hardbottom (brown polygons). Yellow dashed boxes denote shelf-edge “hot-spot” areas off Texas-Louisiana (B), including the Flower Garden Banks National Marine Sanctuary (FGBNMS; red borders) and adjacent banks, as well as Pinnacles region off the Mississippi-Alabama shelf (C).

Average size of female 50% (39.9 ± 18.2 km^2^) and 95% (202.3 ± 86.5 km^2^) KDEs were, on average, twice that of males (18.7 ± 8.65 km^2^ and 91.5 ± 43.6 km^2^). However, statistical analyses showed that KDEs were strongly influenced by the transmission days covariate (*F*_1,37_ = 13.72; *P* = 0.001) and not the predictors sex (*F*_1,37_ = 0.01; *P* = 0.926) or fork length (*F*_1,37_ = 1.14; *P* = 0.293). Polygon overlap analysis of 50% KDEs revealed two distinct regions of intensive use (i.e., join polygon counts >5; Fig 7A). The first occurred along the shelf-edge region between Louisiana and Texas, the core of which (>7 individuals; Fig 7B) encompassed major hardbottom habitats of the Flower Garden Banks National Marine Sanctuary (FGBNMS) (West and East Flower Garden Banks) and Coffee Lump. Additionally, considerably high overlap was observed above several additional banks and Habitat Areas of Particular Concern designated by the National Oceanographic and Atmospheric Administration such as Stetson Bank (also part of FGBNMS), Appelbaum Bank, Claypile Bank, MacNeil Bank, 29 Fathom Bank, Rankin Bank, 28 Fathom Bank, and Geyer Bank. The core area had a largely east-west distribution, similar to the continental shelf edge contour (i.e., 200 m isobath) of the region.

A second area with considerable overlap of 50% KDEs was the Pinnacles region off the edge of the Mississippi-Alabama shelf (Fig 7C). It is worth noting that this region was also adjacent to an area of significant tagging efforts (>10 individuals). While these hardbottom habitats did not intersect with the same join count as in the western GoM, many were apparently used by >5 individuals. This core region had a northeast to southwest distribution, similar to the continental shelf edge contour (i.e., 200 m isobath) of the region.

The various 50% KDEs also overlapped with 2,504 oil and gas platforms ranging from Texas to Alabama. Of this total, 1,756 were from the Louisiana-Texas shelf (western region) and 748 were from the Mississippi-Alabama shelf (eastern region). The majority of platform join counts (77% west, 22% east) intersected with single individual KDEs. However, there were approximately 32 platforms in the western region (1.8%) and 3 (0.4%) from the east that were situated in some of the highest use areas, with join counts as high as 7.

## Discussion

Our findings provide the first insights into the movements and habitat use of tiger sharks across life-stages within an important, yet understudied, portion of their range. Previous investigations into tiger shark horizontal movements in the western North Atlantic Ocean have been restricted primarily to males [4] or females [23] separately, in disparate locations. By simultaneously tracking many males and females of varying life stages within the same region, we observed sex and size-specific differences in distribution and movement rates, as well as associations with large-scale habitat features. While we encountered limitations due to sample size (i.e., comparatively few adult sharks tagged), these data help address knowledge gaps identified for this species [17], as well as provide baseline information to support future studies in the GoM region.

### Spatiotemporal patterns in movement and distribution

Our work documented both ontogenetic and seasonal patterns in tiger shark distribution in the GoM that are consistent with observations from other subtropical systems [48]. Individuals transition from primarily shelf-based lifestyles when <200 cm FL, to increased use of continental slope and deepwater habitats by intermediate sizes, and the highest use of slope habitats by the largest individuals. Additionally, the duration of these transitions appears to be related to ontogeny. Of the few small sharks that left the shelf, most only did so periodically over a few months in spring and summer. Medium-sized sharks that left the shelf initiated this behavior beginning in August, and gradually moved offshore from September through December, after which they slowly returned to shelf waters from February through April. Large adults, on the other hand, made rapid, pronounced offshore migrations in October, with most remaining off the shelf until April. These inshore-offshore patterns in seasonality are consistent with nearshore landings from recreational fishers off the Texas coast, who report the highest occurrence of large tiger sharks in summer followed by a disappearance in fall [40]. Additionally, the cross-basin movements within the GoM appear somewhat consistent with findings from mark-recapture tagging [49], although we found no evidence of GoM departure from the two individuals tracked beyond a year, suggesting a portion of sharks are resident in this large marine ecosystem.

The increased offshore habitat use and rapidity of inshore-offshore transitions observed with ontogeny is likely facilitated by the ability of large individuals to achieve higher rates of movement, as reported elsewhere [4]. This may be due to changes in caudal fin morphology with size, which increases in symmetricity and likely improves long-distance swimming performance [50]. These changes in distribution coincide with life-stage specific resource needs, such as food maximization in juveniles or reproductive opportunities as adults. For example, since they are rapidly growing and require access to consistent food resources early in life, young tiger sharks may benefit from living principally on the GoM shelf where primary productivity and prey densities are considerably higher than deeper depths and/or more consistently encountered [51,52].

Conversely, larger juvenile and adult sharks may venture more frequently off-shelf to partake in seasonal migrations or to access unique and ephemeral food resources that may arise in open-ocean habitats such as sea turtles [4]. Although the northwestern GoM shelf is generally characterized by a persistent western boundary current, simulations from climatological data indicate that several regions along the shelf edge become seasonally conducive to cross-shelf exchange [53]. Periodically, this may help animals from the shelf access deeper water environments. These slope habitats were most frequented by large adult sharks, and may be indicative of orientation with seasonally variable currents in the region [54] in order to find conspecifics for mating, or areas of dense prey. Regardless, the increased use of offshore waters as adults is consistent with other regions. For example, dietary and stable isotope analyses on samples of tiger sharks captured off South Africa demonstrated the increased reliance of this species on offshore waters with increasing size, coinciding with a more pelagic and variable diet [55]. Off eastern Australia, males are apparently absent from nearshore shark catches at the onset of maturity, which has been associated with a transition to deeper offshore waters at this life stage [17]. As such, these offshore excursions may represent preparatory movements for more of an off-shelf lifestyle typical of adults [19].

While tiger sharks are well known for their low at-vessel mortality [56–59], extraordinarily high fecundity [60], and rapid growth rates [61], the wide-ranging distribution of this species can be challenging to fisheries management. For example, our findings add to those of Rooker et al. [39] by identifying that individuals using the high-seas territory outside of the U.S. Exclusive Economic Zone were immature. Juvenile presence means that individuals may be exploited in this unregulated region prior to reproducing, which could have population-level consequences for the species despite its resilience to capture stress and its reproductive potential.

### Large-scale habitat features

Despite strong characterization of bony fish assemblages along the northwestern GoM shelf-edge banks [62,63] and the FGBNMS [64], there have been limited studies on sharks in this region. The discovery of high overlap in tiger shark core ranges along two shelf-edge systems in the GoM highlights the potential influence of these features on the species’ distribution as recently demonstrated for scalloped hammerhead sharks [30]. The only study to document use of shelf-edge banks by tiger sharks in the GoM was conducted by Childs [65], who compiled visual observations from divers and boaters around the FGBNMS and adjacent area. Childs [65] reported the species as present between December and March, with sightings that were either females or an unconfirmed sex across a range of sizes (100–400 cm, total length, TL, which would be approximately 74–335 cm FL). Our results support these previous findings as four of five individuals detected within the boundaries of the sanctuary were females (102–233 cm FL) and observed during the months of January (n=1), March (n=2), April (n=2), May (n=1), September (n=1), and December (n=2). The only male to be detected within the FGBNMS boundaries was mature, and detected in December. Female presence at the FGBNMS, and at times in groups as large as 5 individuals [65], could be indicative of an aggregation site [66] of mixed life stages as has been recently demonstrated for Tiger Beach, Bahamas [67]. A similar function has been suggested for Sackett Bank, a similar habitat in the GoM region known support aggregations of female dusky shark [31]. Childs’ [65] observations of female sharks in the 300-400 cm TL (i.e., 249–335 cm FL) suggests that mature individuals also use the FGBNMS, which overlaps with one of the two putative tiger shark pupping regions along the GoM shelf proposed by Driggers et al. [28], one of which (93−95°W) is located inshore of the FGBNMS. These findings may also explain the higher rates of movement observed in females compared to males, which may exhibit fidelity to these sites and require rapid seasonal migrations to return to them. Together, these findings suggest shelf-edge banks of the northwestern GoM are used by female tiger sharks during cooler months of the year; however, additional tagging of males is needed as only 3 were tagged (compared to 9 females) in the region. Despite these limitations, the data herein suggest shelf-edge habitats may be related to reproduction (i.e., female aggregation sites) for multiple large shark species in the GoM.

Oil and gas platforms were also in the core habitat of tiger sharks in this study. These structures, which span the offshore region from Texas to Alabama, comprise one of the largest unintended artificial reef complexes in the world [68] that are well-known for their capacity to support exceptionally high fish densities [69], and are indeed used by multiple shark species [70]. There are considerable fishery activities associated with production platforms, including recreational hook-and-line (bottom fishing, surface trolling) and commercial vertical longline for a suite of target snapper-grouper species [71]. Tiger shark associations with these structures are not yet well understood, although two individuals tagged in this study (Shark-24 and 25) were collected within a few hundred meters of a production platform, and a recent study confirmed this species interacts with the base of these structures along the continental slope [72]. Such affinities may expose tiger sharks to blowouts similar to Deepwater Horizon, which impacted surface and bottom waters of the north-central GoM region where we documented significant core habitat use [73]. Uptake and trophic transfer of petroleum-based pollutants, which accumulate in sharks [74,75] and are at significantly higher levels in sediments adjacent to platforms [76], needs further research in order to clarify the potential impacts of these anthropogenic activities on tiger shark health and biology.

Long-term and integrative sampling approaches may help further reveal the extent of tiger shark associations with both natural and artificial habitats. Such data can reveal the importance of these habitats over multiple years, and how residency within these systems changes with sex and size class. We suggest future studies incorporate technologies such as acoustic telemetry, which does not require animals to bear large external transmitters or to surface, and has already been applied to tiger sharks elsewhere [25,77,78]. Such data would also provide a glimpse into inter-bank and platform connectivity, site fidelity, and the role of tiger sharks in transporting nutrients across these various sites (i.e., allochthonous inputs). Additionally, given the relatively high water clarity of the FGBNMS and other banks in the region, an integrated baited remote underwater video approach may also help reveal association patterns and size structure of tiger sharks from these habitats, as done recently in the Galápagos Marine Reserve [79].

### Study limitations and recommendations

Our tagging efforts uncovered some potential limitations in the application of satellite telemetry to tiger sharks, particularly with respect to shark size. That is, we found that tags affixed to multiple individual sharks <200 cm FL did not transmit. Additionally, of those that transmitted, none were tracked beyond 60 days. While post-release mortality is possible, this is unlikely given that previous research has revealed that tiger sharks are relatively robust to capture stress, exhibiting high at-vessel and post-release survival in experimental studies [56–58].

One explanation for transmission failure could be the relatively smaller sized and thin first dorsal fin of young tiger sharks, and thus reduced potential for the fin to stay erect when above water due to the gravitational pull on the tag. Males and females grow at rapid rates (20-40 cm·yr^-1^) until they reach 200–250 cm FL [80]. How these changes in growth rate translate to first dorsal fin shape has not yet been quantified, although some morphological differences are likely between adults and juveniles [50]. These higher growth rates may also encapsulate the SPOT transmitter within animal tissue, preventing wet-dry sensors from operating normally, or alternatively may eject the transmitter as a foreign body [81,82].

A more likely explanation for lower transmission rates at smaller sizes is behavioral, namely that smaller animals simply spend less time at the surface than larger individuals. Younger individuals in the GoM are reported to forage disproportionately on benthic gastropods [83], whereas adults, which are known to spend the majority of their time at depths <5 m [48,84], more commonly consume sea turtles and fishes [83]. Additionally, smaller individual tiger sharks are likely more susceptible to predation from larger sharks and as such may avoid these areas at younger life stages to improve survival. Regardless, given the expansion of coastal acoustic tracking arrays along the continental shelf, acoustic telemetry approaches may be a preferred way to track migrations and habitat use of younger tiger sharks in the GoM.

**Fig 8:**
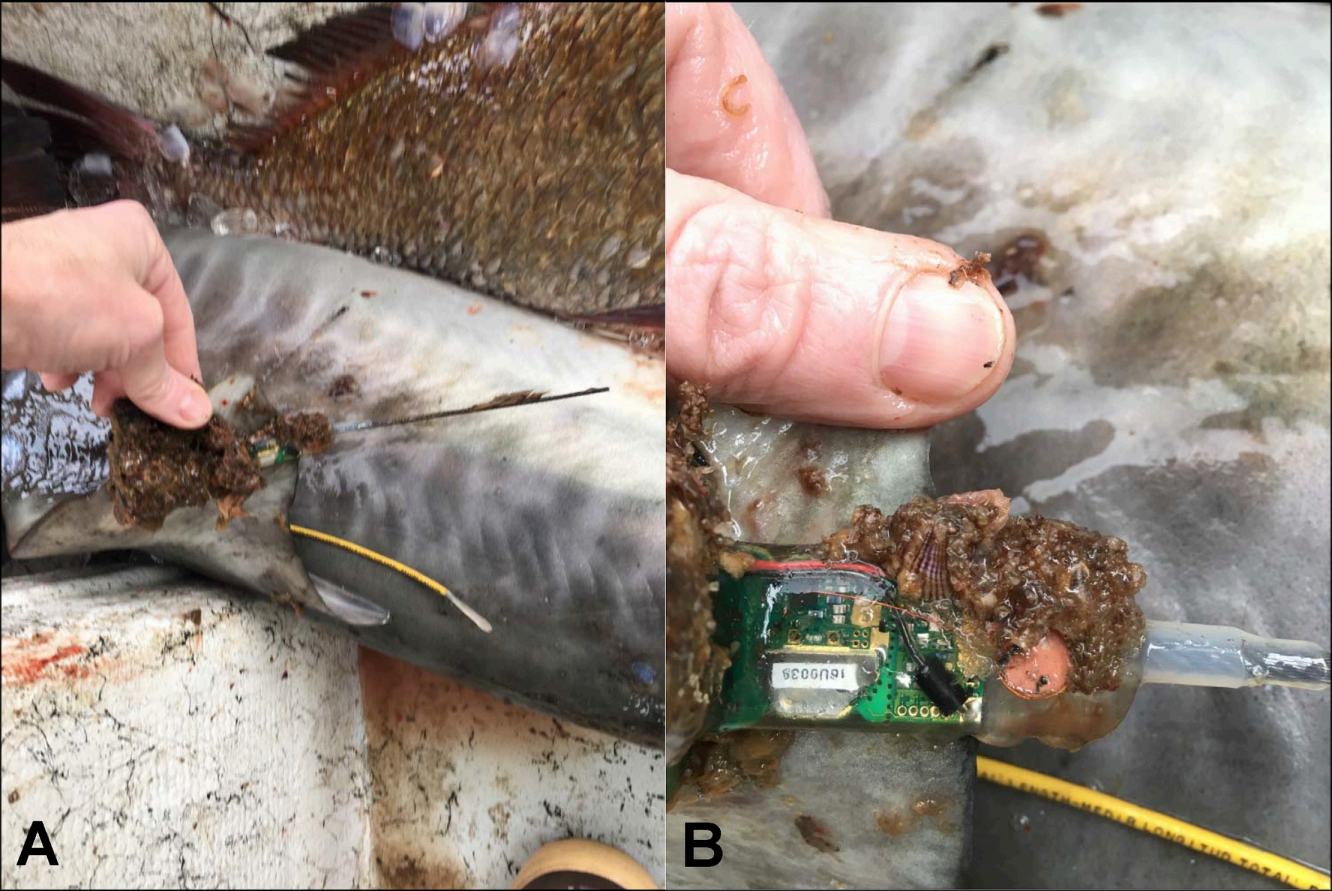
Photographs of a recaptured shark and transmitter. Photographs indicate biofouling on Wildlife Computers SPOT 258-A from Shark-45 after 7 months at liberty. Photos show an overview of the tag and relative placement on the dorsal fin (A), and a closeup of the wet-dry sensors and other internal components (B).

The only sharks to surpass one year of tracking duration were programmed with a maximum rate of 70 transmissions per day, whereas all other tags were programmed with the default rate of 250 transmissions per day. As such, battery exhaustion may have played a role in the overall longevity of the tags. The SPOT tags from these two individuals were also coated in black antifouling paint (Interlux, inc.). Although it has not been demonstrated experimentally, biofouling purportedly decreases transmission rates and potentially track durations [85]. Indeed, Shark-45 (141 cm FL) was fitted with a SPOT in May 2016, yet never transmitted position estimates. This individual was captured by a collaborator in December 2016, 67 km from the release site. Although this tag was coated in clear antifoulant, after being at large for seven months all sensors on the tag were severely biofouled (Fig 8). While the general lack of transmissions for this individual is also likely behavioral as mentioned above, highly biofouled tags have been reported previously for the species [86], and highlight technical limitations to be considered when using transmitters in productive coastal environments like portions of the GoM. Unfortunately, we are unable to isolate the source of variation for tag performance due to lack of replication and a variety of confounding factors (tagging location, antifoulant, transmission cycles, animal size). Further research into ways to increase the longevity of these transmitters is thus warranted, and could benefit from balanced and replicated experimental designs.

## Conclusions

The present study examined ontogenetic and sex-related movement and distribution patterns of tiger sharks within the GoM. These data provide a baseline for comparison against, and/or predicting their vulnerability to, future environmental change, such as climate variability or oil spills. Future research can benefit from combining alternative tracking approaches that facilitate long-term assessment of the species’ ecological dynamics, particularly for the vulnerable young life stages. Further, additional tracking efforts are needed for adults, which were highly underrepresented in this data set and thus limit inferences on mating and parturition grounds. The revelation of core habitat use areas encompassing National Oceanographic and Atmospheric Administration designated Habitat Areas of Particular Concern, where anthropogenic activities are restricted, as well as highly modified environments such as artificial reefs and heavily exploited nearshore habitats, demonstrates the complex across-shelf habitat connectivity exhibited by tiger sharks and potentially other large mobile predators in this highly dynamic system.
